# *Vibrio parahaemolyticus*: a review on the pathogenesis, prevalence, and advance molecular identification techniques

**DOI:** 10.3389/fmicb.2014.00705

**Published:** 2014-12-11

**Authors:** Vengadesh Letchumanan, Kok-Gan Chan, Learn-Han Lee

**Affiliations:** ^1^Jeffrey Cheah School of Medicine and Health Sciences, Monash UniversityBandar Sunway, Malaysia; ^2^Division of Genetics and Molecular Biology, Institute of Biological Sciences, Faculty of Science, University of MalayaKuala Lumpur, Malaysia

**Keywords:** *Vibrio parahaemolyticus*, food borne, prevalence, pathogenesis, virulence factors, clinical manifestation, molecular techniques

## Abstract

*Vibrio parahaemolyticus* is a Gram-negative halophilic bacterium that is found in estuarine, marine and coastal environments. *V. parahaemolyticus* is the leading causal agent of human acute gastroenteritis following the consumption of raw, undercooked, or mishandled marine products. In rare cases, *V. parahaemolyticus* causes wound infection, ear infection or septicaemia in individuals with pre-existing medical conditions. *V. parahaemolyticus* has two hemolysins virulence factors that are thermostable direct hemolysin (*tdh*)-a pore-forming protein that contributes to the invasiveness of the bacterium in humans, and TDH-related hemolysin (*trh*), which plays a similar role as *tdh* in the disease pathogenesis. In addition, the bacterium is also encodes for adhesions and type III secretion systems (T3SS1 and T3SS2) to ensure its survival in the environment. This review aims at discussing the *V. parahaemolyticus* growth and characteristics, pathogenesis, prevalence and advances in molecular identification techniques.

## INTRODUCTION

The presence of pathogenic bacteria in the worldwide marine environment raises concerns of human on food safety due to the latter potentially causing disease outbreaks depending on the environmental conditions (Ceccarelli et al., 2013). A good example is *Vibrio parahaemolyticus*, a member of *Vibrio* species from the Vibrionaceae family. *V. parahaemolyticus* is a Gram-negative halophilic bacterium that is widely disseminated in estuarine, marine and coastal surroundings (Su and Liu, 2007; Nelapati et al., 2012; Ceccarelli et al., 2013; Zhang and Orth, 2013). *V. parahaemolyticus* is usually found in a free-swimming state; with its motility conferred by a single polar flagellum affixed to inert and animate surfaces including zooplankton, fish, shellfish or any suspended matter underwater (Gode-Potratz et al., 2011). The classification of *V. parahaemolyticus* depends on the antigenic properties of the somatic (O) and capsular (K) antigen produced in various environmental condition (Nair et al., 2007).

*Vibrio parahaemolyticus* was first discovered by Tsunesaburo Fujino in 1950 as a causative agent of food borne disease following a large outbreak in Japan which recorded 272 illnesses with 20 deaths after consumption of *shirasu*. (Fujino et al., 1953). Virulent *V. parahaemolyticus* strains are transmitted by consumption of raw or undercooked seafood causing acute gastroenteritis (Newton et al., 2012; Zarei et al., 2012). In rare cases, *V. parahaemolyticus* causes wound infection, ear infection or septicaemia that may be life-threatening to individuals with pre-existing medical conditions (Zhang and Orth, 2013). Since its discovery, *V. parahaemolyticus* has been found to be responsible for 20–30% of food poisoning cases in Japan and seafood borne diseases in many Asian countries (Alam et al., 2002). *V. parahaemolyticus* was also recognized as the leading cause of human gastroenteritis associated with seafood consumption in the United States (Kaysner and DePaola, 2001; Newton et al., 2012).

The worldwide prevalence of *V. parahaemolyticus* gastroenteritis cases stresses the need for understanding of the virulence factors involved and their effects on humans. This article aims to discuss on *V. parahaemolyticus* as an emerging pathogen associated with seafood consumption and its effects to human in terms of pathogenesis, prevalence and the advances in molecular identification techniques used to identify *V. parahaemolyticus*.

## PATHOGENESIS OF *Vibrio parahaemolyticus*

*Vibrio parahaemolyticus* strains have a number of different virulence factors including adhesins, thermostable direct hemolysin (*tdh*) and TDH related hemolysin (*trh*) as well as two type III secretion systems, T3SS1 and T3SS2 (Makino et al., 2003). *V. parahaemolyticus* strains are encoded with T3SS1 to ensure its survival in the environment (Paranjpye et al., 2012). The T3SS1 have a number of virulence factors that cause lysis of an infected host cell and allow for the release of important nutrients (Burdette et al., 2008). In addition, some *V. parahaemolyticus* strains gain a T3SS2, and *tdh* and TDH related hemolysin (*trh*) genes which lead to a number of strains with different degrees of pathogenicity. Besides T3SSs and TDH genes, *V. parahaemolyticus* have two different types of flagella with distinct functions for swimming and swarming, as well as the ability to produce a capsule. Both these factors are likely to help in the strains survival in the environment and also in colonization of a human host (Broberg et al., 2011).

### ADHESION TO HOST CELLS

The most important step in bacterial pathogenesis is initial host cell binding. During infection, bacterial adhesion factors are present at the bacterial surface to form contact with host cell for secretion of effectors and toxin proteins. MAM7 (Multivalent Adhesion Molecule 7) is a novel adhesion which is conserved in many Gram-negative bacteria. MAM7 consists of a hydrophobic stretch of 44 amino acids at its N terminus, which is required for correct localization and outer membrane anchoring of the protein. MAM7 also contains seven mammalian cell entry (mce) domains (Zhang and Orth, 2013). MAM7 is constitutively expressed, enabling Gram-negative pathogens to establish immediate contact with host cells upon their first encounter, which in turn can lead to up-regulation of other pathogen-specific or host cell-specific adhesion and virulence factors (Krachler and Orth, 2011).

In the process, MAM7 will bind to both fibronectin and phosphatidic acid, and if either of these substrates is blocked, it could prevent adhesion of MAM7 to host cells. Heterologous expression of MAM7 is sufficient for attachment of a non-pathogenic *Escherichia coli* strain to host cells. This could in turn block attachment and attenuate cytotoxicity of *V. parahaemolyticus* or any other MAM7-expressing Gram-negative pathogens. In addition, MAM7 is necessary for initial host binding during infection and for T3SS-mediated cell death in some cell types. These insight on MAM7 provide a new perspective on bacterial and host cell interactions (Krachler et al., 2011). Furthermore, the discovery of MAM7 led to new research investigating this molecules potential as a therapeutic agent for many Gram-negative bacteria including *V. parahaemolyticus* (Krachler et al., 2012).

### TOXINS

Outbreaks of *V. parahaemolyticus* illness have increased. This is particularly found in countries with high levels of seafood consumption where *V. parahaemolyticus* causes over half of all food-poisoning outbreaks of bacterial origin (Daniels et al., 2000a). The *tdh* and TDH-related hemolysin (*trh*) are the two virulence factors associated with *V. parahaemolyticus* hemolysis and cytotoxicity activity in the host cell (Broberg et al., 2011; Zheng et al., 2014). *V. parahaemolyticus* bacteria are extensively present in marine and estuarine environments but not all strains of this bacterium are considered pathogenic (Velazquez-Roman et al., 2012). The strains isolated from environmental samples usually lack the pathogenic genes *tdh* and/or *trh* which cause illnesses to humans and marine animals (Deepanjali et al., 2005; Canizalez-Roman et al., 2011; Gutierrez West et al., 2013). Nevertheless, studies from U.S., Europe and Asia have reported around 0–6% of the environmental samples analyzed to be positive for the presence of *V. parahaemolyticus* strains with *tdh* gene and/or *trh* genes (Kaysner et al., 1990; DePaola et al., 2000; Vuddhakul et al., 2000; Wong et al., 2000a; Alam et al., 2002; Hervio-Heath et al., 2002).

Commonly, all the clinical *V. parahaemolyticus* strains isolated from humans with gastroenteritis are differentiated from the environmental strains based on the strains ability to produce *tdh* which can lyse red blood cells on Wagatsuma blood agar. This hemolytic activity on Wagatsuma agar is known as Kanagawa phenomenon (Nishibuchi et al., 1989; Alipour et al., 2014). Only 1–2% of the environmental samples is reported to be KP-positive and the rest are categorized as KP-negative strains (Nishibuchi and Kaper, 1995; Alipour et al., 2014). Molecular epidemiological studies report that *Vibrio parahaemolyticus* KP-negative strains did not feature *tdh* gene characteristic but produce a *trh* gene. A study has reported the isolation of a KP-negative *V. parahaemolyticus* strain that produces *trh* gene from an outbreak of gastroenteritis in the republic of Maldives in 1985 (Qadri et al., 2005). The *trh* gene plays a role similar to *tdh* gene in the pathogenesis of *V. parahaemolyticus* and is therefore considered a virulence factor of *V. parahaemolyticus* (Nelapati et al., 2012).

### TYPE III SECRETION SYSTEMS OF *Vibrio parahaemloyticus*

Type III secretion systems are needle-like bacterial machinery used to inject bacterial protein effectors directly into the membrane and cytoplasm of eukaryotic cells without encountering with the extracellular environment (Cornelis, 2006). T3SSs is made up of 20–30 proteins with a secretion apparatus consisting of a basal body that spans the inner and outer bacterial membranes, a needle that is polymerized and extended into extracellular space, and a translocon pore that is inserted into the eukaryotic cell membrane (Izore et al., 2011). Some secretion apparatus proteins have homology to flagellar export proteins, with core transmembrane proteins showing the highest level of conservation (Marlovits and Stebbins, 2010). The common targets of T3SS effectors include the actin cytoskeleton, innate immune signaling, and autophagy. Depending on the pathogens needs, the systems can be either up regulated or down regulated (Broberg et al., 2011).

#### T3SS1

The T3SS1 is present in all environmental and clinical *V. parahaemolyticus* strains (Paranjpye et al., 2012). This system is similar to *Yersiniaysc* T3SS in terms of the number of genes, its gene identity and the characteristic of being induced by increasing temperature or decreasing the calcium concentration (Ono et al., 2006; Zhang and Orth, 2013). The T3SS1 system is regulated by three interacting proteins (ExsC, ExsD, and ExsE) that control the activity of the master transcriptional regulator ExsA, a member of AraC family of transcription activators. Under non-inducing conditions, ExsA is bound to ExsD and rendered inactive, while ExsC, an anti-anti-activator of the system, is bound to ExsE. Under inducing conditions, when ExsE is secreted, ExsC is released and binds to ExsD which allows the release of ExsA and activates the transcription of T3SS1 genes (Zhou et al., 2010).

During tissue cell infection, T3SS1 initiates a series of events that involves autophagy, membrane blebbing, cell rounding, and lastly cell lysis. This entire reproducible series of events is carried out by three main effectors from T3SS1 gene including VopQ (VP1680), VPA0450, and VopS (VP1686). VopQ is induced by Pi3-kinase independent autophagy upon infection with POR3 or transfection of VopQ into Hela cells and prevents phagocytosis of the infecting bacteria (Sreelatha et al., 2013). In the event the VopQ is absent, infected macrophages are able to phagocytose *V. parahaemolyticus* and induce apoptosis. Thus, sequestering membrane resources by inducing autophagy is proposed to antagonize the ability of host cells to phagocytose *V. parahaemolyticus*.

VPA0450 is an inositol polyphosphate-5-phosphatase that hydrolyzes the D5 phosphate from phosphatidylinositol-4,5-bisphosphate [PI(4,5)P2] in the plasma membrane (Broberg et al., 2010). It destabilizes the cell by detachment of the plasma membrane from the actin cytoskeleton, which leads to blebbing of plasma membrane and allows rapid lysis of the host cell (Broberg et al., 2010; Zhang and Orth, 2013). The third effector is VopS that targets the actin cytoskeleton by AMPylating Rho-family GTpase. It is responsible for the collapse of actin cytoskeleton which leads to cell rounding and shrinkage (Broberg et al., 2010; Ceccarelli et al., 2013). The Fic domain within VopS mediates the direct transfer of adenosine monophosphate from ATP to the switch 1 region of these small Gproteins, which prevents their binding to downstream effectors. This blocks the signaling cascade regulating the actin cytoskeleton and lead to its collapse (Zhou et al., 2010).

#### T3SS2

The T3SS2 encoded on a pathogenicity island on chromosome 2 is found in clinical isolates and is associated with pandemic *V. parahaemolyticus* strains (Paranjpye et al., 2012). T3SS2 is different from T3SS1, but has closest homology to the Hrp1 system found in *Pseudomonas syringae* (Park et al., 2004; Cornelis, 2006). In a rabbit ileal loop model, T3SS2 effectors are translocated into host cells causing cytotoxicity of colon epithelial and enterotoxicity within the host (Park et al., 2004). T3SS2 is closely associated with pathogenicity island (VPAI-7) and flanked by two *tdh* genes (Zhang et al., 2012). The effectors of T3SS2 include VopC (VPA1321), VopT (VPA1327), VopA/P (VPA1346), and VopL (VPA1370).

VopC (VPA1321) has homologies to cytotoxic necrotizing factor 1 (CNF1), an exotoxin that is described in pathogenic *E. coli* strains. CNF1 activates Rho, Rac, and Cdc42 by deamidating a glutamine residue in the switch 2 region of each enzyme and preventing hydrolysis of GTP. This induces changes in the actin cytoskeleton and facilitates *V. parahaemolyticus* entry into non-phagocytic host cells (Zhang et al., 2012). The second effector is VopT (VPA1327), an ADP ribosyltransferase domain of the *P. aeruginosa* effectors ExoS and ExoT, which is able to modify the small GTPase Ras (Kodama et al., 2007). The VopT gene’s is activity is partially responsible for the cytotoxicity seen during infection of Caco-2 monolayers with *V. parahaemolyticus* (Kodama et al., 2007).

VopA or also known as VopP is categorized as a YopJ homolog that blocks activation of the MAPK signaling pathway by acetylating a conserved serine, threonine, and lysine residue on MAPKKs. This prevents the phosphorylation and activation of the MAPK pathway, which prevents the induction of cytokines (Trosky et al., 2004). The activity of VopA/P could possibly be partially redundant with VopT as both block the activation of the ERK/MPK pathway (Broberg et al., 2011). The VopL (VPA1370) is a protein containing three Wiskott–Aldrich homology 2 (WH2) domains. This effector is responsible for the strong actin filament nucleation which is observed in host cell (Namgoong et al., 2011).

### TYPE VI SECRETION SYSTEMS (T6SS1 AND T6SS2)

The type VI secretion systems, T6SS1 (VP1386–VP1420) and T6SS2 (VPA1030–VPA1043) are located on chromosome 1 and 2 respectively on *V. parahaemolyticus* RIMD 2210633 (Boyd et al., 2008; Izutsu et al., 2008). Preliminary data suggested that the T6SS2 is an adhesion and not involved in cytotoxicity (Yu et al., 2012). Since T6SS2 and T3SS2 systems co-exist, it is suggested that both systems might cooperate during an infection on host. T6SS2 takes the first step of infection as a role of adhesion where else T3SS2 exports effectors by inducing enterocytotoxicity (Park et al., 2004; Yu et al., 2012). It is reported that T6SS gene was used as a virulence marker to differentiate pandemic and non-pandemic strains isolated in Japan. The T6SS gene was present in all pandemic strains, whereas majority of the non-pandemic strains had a partial set of T6SS genes (Ceccarelli et al., 2013). In addition, researchers have reported that T6SS1 is most active under warm marine-like conditions, while T6SS2 is active under low salt conditions and that surface sensing and quorum sensing differentially regulate both systems (Salomon et al., 2013).

### PANDEMIC CLONE OF *Vibrio parahaemolyticus*

Gastroenteritis due to *V. parahaemolyticus* occurs as sporadic cases caused by different serotypes. The epidemiology of this bacteria changed with the emergence of pandemic clone of 03:K6 serotype in Kolkata, India in year 1996 (Bisha et al., 2012). Since then, this 03:K6 strains have been involved in many food borne outbreaks in Asian countries (Matsumoto et al., 2000), United States and worldwide. Currently there are more than 20 serovariants including 03:K6, 04:K68, 01:K25, and 01:KUT (Nair et al., 2007).

The clinical isolates of pandemic and non-pandemic *V. parahaemolyticus* showed the presence of a 24 kb region which is known as *V. parahaemolyticus* island-1 (Vp-PAI-1) in 03;K6. Further molecular analysis on the other genomic islands demonstrated the presence of Vp-PAI-4, Vp-PAI-5, and Vp-PAI-6 in the pandemic strains (Hurley et al., 2006). The Vp-PAI-1 is suggested to be one of the markers of pandemicity owing to the presence of a virulence gene (Nishioka et al., 2008). The isolates in the pandemic group carried *tdh* gene but not *trh* gene, *orf8*, Vp-PAI-1, Vp-PAI-5, Vp-PAI-7, and T3SS2, while the non-pandemic isolates are heterogeneous (Chao et al., 2009). The pandemic 03:K6 strains were detected with *toxRS* sequence which was useful to differentiate between pandemic and non-pandemic *V. parahaemolyticus* strains (Matsumoto et al., 2000). The differences studied among and between 03:K6 strains led to the definition of non-pandemic 03;K6 strains isolated in 1980–1990 in Asian countries including India, Taiwan, Japan, Thailand, and Bangladesh (Ceccarelli et al., 2013).

Literature stated the presence of filamentous phage *f237* in a many 03:K6 isolates which suggests a specific association between the phage and widespread of 03:K6 serotype (Nasu et al., 2000). *V. parahaemolyticus* 03:K6 strains also have *orf8* located in the phage and encoding a putative adherence protein which may have played important role in increasing the virulence of 03:K6 isolates by being more adhesive to host intestinal cells (Ceccarelli et al., 2013). The genetic traits have been suitable markers for pandemic strains identification however there are inconsistencies noted whereby pandemic O3:K6 strains with atypical profiles isolated in Taiwan, Bangladesh, Japan, and Thailand (Jones et al., 2012).

## PREVALENCE OF *Vibrio parahaemolyticus*

Seafood is a nutrient rich part of a healthy diet and seafood consumption is associated with various health benefits (Iwamoto et al., 2010). Approximately 90% of global aquaculture production in based in Asia region. However, along with nutritional benefits from seafood consumption come the potential risks of eating contaminated seafood. Seafood is known as a vehicle of transmission of food borne bacteria that cause human illness worldwide. World Health Organization (WHO) defines food borne illness as a disease which is caused by consumption of contaminated food (Velusamy et al., 2010). Pathogens such as *Vibrio* species, *E. coli* 0157:H7, *Campylobacter*, *Salmonella*, and *Listeria monocytogenes* have been found to be responsible for major food borne outbreaks worldwide (Apun et al., 1999; Velusamy et al., 2010). In the Asian region, *Vibrio* species have been recognized as the leading cause of food borne outbreaks in many countries including Japan, India, China, Taiwan (Hara-Kudo et al., 2003), Korea (Lee et al., 2008), and Malaysia (Tunung et al., 2010). Outbreaks in Asia were reported to be mainly caused by consumption of contaminated seafood (Jacxsens et al., 2009).

In the Asian region, *V. parahaemolyticus* was first recognized as a food borne pathogen in the year 1951 in Osaka, where people frequently consume raw or uncooked seafood (Daniels et al., 2000b). The bacterium was isolated from victims of an outbreak of 272 infected cases and 20 deaths associated with consumption of shirasu, Japanese boiled and semi dried sardines dish (Aberoumand, 2010). Ever since then, *V. parahaemolyticus* has been commonly isolated from seafood, including shrimp, in markets in South East Asian countries (Elhadi et al., 2004; Deepanjali et al., 2005). *V. parahaemolyticus* has accounted for many food poisoning cases in Japan (Su and Liu, 2007; Aberoumand, 2010; Kubota et al., 2011; Hara-Kudo et al., 2012), in Taiwan (Wong et al., 2000b; Anon, 2005; Yu et al., 2013), in China since early 1990s (Jiang, 1991; Wu et al., 1998; Liu et al., 2004; Li et al., 2014), Bangladesh (Bhuiyan et al., 2002), Laos (Matsumoto et al., 2000), Hong Kong, and Indonesia (Matsumoto et al., 2000).

Pathogenic *V. parahaemolyticus* was also isolated in Thailand, which is the primary producer and exporter of cultured shrimp worldwide (Yano et al., 2014). A recent study reported the presences of antimicrobial resistance *V. parahaemolyticus* isolates from white leg shrimp and black leg shrimp cultured at inland ponds in Thailand (Yano et al., 2014). Besides Thailand, pathogenic and antimicrobial resistance *V. parahaemolyticus* was also isolated from shrimps and cockles in Malaysia (Al-Othrubi et al., 2011). Moreover, in recent years, *V. parahaemolyticus* has been reported as one of the leading cause of food borne diseases in China (Liu et al., 2004; Chen et al., 2010). Literature has shown that the retail foods in Chinese markets are contaminated by various food borne pathogens such as *L. monocytogenes* (Chen et al., 2014), *Salmonella spp.* (Chen et al., 2013), and *Campylobacter jejuni* (Zheng et al., 2014), and *V. parahaemolyticus*. In addition, shrimp contaminated with *V. parahaemolyticus* has been associated with outbreaks of food borne illnesses in China (Peng et al., 2010). A recent study reported the isolation of pathogenic *trh V. parahaemolyticus* from shrimps with the bacteria densities less than 100 MPN/g in samples (Xu et al., 2014).

In India, *V. parahaemolyticus* has been isolated both from clinical and environmental samples. In a recent clinical study, 178 *V. parahaemolyticus* strains were isolated from 13,607 diarrheal patients admitted in Infectious Diseases Hospital, Kolkata since 2001 to 2012 (Pazhani et al., 2014). *V. parahaemolyticus* diarrheal cases were also detected from the urban slums of Kolkata, India (Kanungo et al., 2012). Reyhanath and Kutty (2014) have reported the detection and isolation of multidrug resistant strains of *V. parahaemolyticus* from a fishing land at South India. In another study, pathogenic and antibiotic resistant *V. parahaemolyticus* strains along with other *Vibrio* species strains were isolated from seafood in Cochin. Majority of the strains in this study were resistant to ampicillin and multiple drug resistance was prevalent among the isolates (Sudha et al., 2014). In Taiwan, a study reported the isolation of *V. parahaemolyticus* from oysters and clam at culturing environments. The isolates exhibited hemolytic or urease activities and presence of *tdh* gene, *trh* gene and T3SS (Yu et al., 2013). The occurrence of pathogenic *V. parahaemolyticus* in seafood and its antimicrobial resistance profile is of public health concern which demands immediate attention.

In Europe, *V. parahaemolyticus* has been isolated from the Baltic Sea, the North Sea, the Mediterranean Sea (Miwatani and Takeda, 1976), and the Black Sea (Aldova et al., 1971). In 1978, studies were conducted in coastal waters of Guadeloupe and *V. parahaemolyticus* was isolated from 53 of 100 water samples investigated (Papa, 1980). As years passed, numerous cases of *V. parahaemolyticus* gastroenteritis were detected and isolated in Spain, Greece, Britain, Turkey, Denmark, Yugoslavia, and the Scandinavian areas (Qadri et al., 2005). A serious outbreak affecting 44 patients associated with consumption of shrimps imported from Asia occurred in France in 1997 (Robert-Pillot et al., 2004). In 1999, there was an outbreak in Galicia, Spain of 64 cases due to consumption of raw oysters (Lozano-Leon et al., 2003). In year 2004, another outbreak was reported in Spain involving 80 cases of *V. parahaemolyticus* infection among wedding guests in a restaurant. The investigation revealed that the outbreak was caused by consumption of boiled crab prepared under unsanitary conditions (Martinez-Urtaza et al., 2005).

In 1971, *V. parahaemolyticus* was first identified as an etiological food borne pathogen in Maryland, U.S. after three outbreaks of 425 gastroenteritis cases associated with consumption of improperly cooked crabs (Molenda et al., 1972). Ever since then, intermittent *V. parahaemolyticus* outbreaks have been reported throughout the U.S. coastal regions due to the consumption of raw shellfish or uncooked seafood. The Centers for Disease Control and Prevention [CDC] (1998) have reported about 40 outbreaks of *V. parahaemolyticus* infection from the year 1973 to 1998 (Daniels et al., 2000a). Four out of the 40 outbreaks involved over 700 cases of diseases linked with consumption of raw oyster in the Gulf Coast, Pacific Northwest, and Atlantic Northeast regions between the years 1997 to 1998. During the summer of 1997, there were 209 (including one death) of *V. parahaemolyticus* infection cases reported involving raw oyster consumption in the Pacific Northwest (Oregon, Washington, California, and British Columbia of Canada; Centers for Disease Control and Prevention [CDC], 1998). Two outbreaks of 43 cases in Washington and 416 cases in Texas in the 1998 were also associated with consumption of raw oyster (DePaola et al., 2000). Another small outbreak of eight cases of *V. parahaemolyticus* illnesses was reported in Connecticut, New Jersey, and New York between July and September in 1998 as a result of eating oysters and clams harvested at Long Island Sound of New York (Centers for Disease Control and Prevention [CDC], 1999). In summer 2004, 14 passengers on board a cruise ship in Alaska manifested gastroenteritis symptoms after ingestion raw oysters produced in Alaska (McLaughlin et al., 2005). The O6:K18 isolates from the Alaskan outbreak were in distinguishable by PFGE from those isolated in the sporadic cases from Pacific Coast states over the previous decade. From July to October of 2004, 96 environmental samples were collected from 17 Alaskan oyster farms, and 31 samples (32%) tested positive for *V. parahaemolyticus*. The most frequently occurring serotypes were O1:K9, O4:K63, and O6:K18 (Newton et al., 2012). In summer 2006, an outbreak occurred involving 177 cases of *V. parahaemolyticus* associated with consumption of contaminated oysters harvested in Washington and British Columbia (Centers for Disease Control and Prevention [CDC], 2006).

Pandemic *V. parahaemolyticus* strains were also isolated in The United States. The O4:K12 serotype showed the highest prevalence among tested clinical *V. parahaemolyticus* isolates from the U.S. Pacific Coast between 1979 and 1995 (DePaola et al., 2003). In 1998, another outbreak occurred involving 416 individuals from 13 states across U.S. after consumption of raw oysters. From the available patients stool samples, *V. parahaemolyticus* O3:K6 was isolated, which closely resembled the pandemic Asian O3:K6 isolates by PFGE (Daniels et al., 2000a). Clinical isolates in the U.S., especially from the Pacific Northwest are found to be encoded with *trh* gene (Paranjpye et al., 2012). In addition, there was an increase in clinical isolates possessing either *tdh* gene, *trh* gene or both, and these severe cases required hospitalization (FAO/WHO, 2011). These incidents of *V. parahaemolyticus* contamination in oysters reflect a serious safety concern in the U.S.

Current studies have reported that environmental factors which include interaction with other hosts play a huge effect in the evolution of certain pathogens (Wilson and Salyers, 2003). Therefore, the pandemic strains that exhibit certain biological characteristics such as increased toxin production or having the capability to live within the natural environment could give better insights into the mechanisms underlying the emergence and spread of these strains (Wong et al., 2000a). The prevalence and distribution of *V. parahaemolyticus* is known to be influenced by several environmental factors including the water temperature, salt and oxygen concentrations, interaction with plankton, presence of sediment, organic matter in suspension and marine organisms (Cabrera-Garcia et al., 2004). Despite the advances in hygiene, food treatment and food processing, this food borne pathogen still represents a significant threat to human health worldwide.

## IDENTIFICATION OF *Vibrio parahaemolyticus*

### ENRICHMENT MEDIA AND SELECTIVITY

*Vibrio parahaemolyticus* is recognized as a cause of food borne illness that is associated with seafood consumptions. Hence, various selective enrichment media have been utilized for the isolation and detection of *V. parahaemolyticus* (Paydar et al., 2013). Due to its natural presence in the marine environments with high tolerance and preference to alkaline pH condition, the selective media used for this pathogen is often prepared for pH 8.6–pH 9.4, alkaline with the additional 1–7% NaCl. In certain condition, the media is supplemented with extra surfactants such as sodium dodecyl sulphate (SDS) and alkylbenzene sulphonate, bile salts, dyes such as metachrome yellow II RD, and antibiotics such as colistin or polymyxin B (Donovan and Van Netten, 1995).

The U.S. Food and Drug Administration (FDA) have recommended alkaline peptone water (APW) as the enrichment broth for all *Vibrio* species including *V. parahaemolyticus* (Farmer et al., 2003; DePaola and Kaysner, 2004). APW has a pH level between pH 8.5–pH 9 and high concentration of NaCl which inhibits the growth of other bacteria (DePaola and Kaysner, 2004). Preparation of this APW, 10.0 g Peptone and 10.0 g NaCl in 1000 ml distilled water. The pH is adjusted to 8.5 ± 0.1 and autoclaved at 121°C for 10 min. Besides APW, salt polymyxin broth (SPB), alternative protein source (APS) broth, salt colistin broth, glucose salt teepol broth and bile salt sodium taurocholate (ST broth) can be used as an enrichment broth for *Vibrio* species (Bisha et al., 2012). In a study, the results stated higher percentage of isolation and identification of pathogenic *V. parahaemolyticus* strains from seafood samples by using ST broth compared to APW (Raghunath et al., 2009). Hara-Kudo et al. (2001) have developed a procedure that consists of a non-selective enrichment step in salt trypticase soy broth followed by a selective enrichment step in SPB. This two step enrichments procedure was found to be more effective to isolate *V. parahaemolyticus* compared to the one step enrichment in SPB alone. SPB contains Polymyxin B sulfate that inhibits the growth of gram-positive organisms.

Various selective media have been developed for isolation and identification of *V. parahaemolyticu*s. The most common selective media is thiosulphate citrate bile salts sucrose (TCBS), a highly selective differential medium that is widely used not only for *Vibrio cholerae* but all other pathogenic *Vibrios* except *Vibrio hollisae* (Kobayashi et al., 1963). TCBS is a selective system consisting of ox bile (0.8%), NaCl (1%) and alkaline pH 8.6 which suppresses the of growth other interfering gram positive organisms. The main advantage of TCBS agar is its sucrose/bromothymol blue diagnostic system which differentiates sucrose-positive *Vibrios* such as *V. cholerae* from other *Vibrio* species colonies. *V. cholerae* would resemble notable colony morphology of 2–3 mm, yellow colonies on TCBS agar (Mrityunjoy et al., 2013). *V. parahaemolyticus* colonies would be typical 2–3 mm diameter, round, opaque, green, or bluish colonies (Bisha et al., 2012).

As year passed, researchers have noted from surveys of seafood studies that *V. parahaemolyticus* colonies on TCBS agar are difficult to distinguish physically from other bacterial colonies. Since TCBS is a general media used for all *Vibrio* isolation, a huge amount of yellow colonies produced by sucrose-fermenting bacteria or green colonies will grow on this media and make it difficult to effectively isolate and enumerate *V. parahaemolyticus* from samples (Pinto et al., 2011; Bisha et al., 2012). To offset this issue, Hara-Kudo et al. (2001) developed a new enrichment procedure and selective agar medium for detecting *V. parahaemolyticus* in seafood. The samples were cultured in selective SPB and plated on the chromogenic CHROMagar *Vibrio* (CV) agar (CHROMagar Microbiology, Paris, France). CHROMagar contains colorimetric substrates for β-galactosidase and was developed specifically to differentiate ortho-nitrophenyl-β-galactoside-positive *V. parahaemolyticus* from other closely related *Vibrio* species (Bisha et al., 2012). On this chromogenic medium, the mauve color *V. parahaemolyticus* colonies are easily distinguished and differentiated from other *Vibrio* species. Many researchers have compared the use of CV and TCBS, and reported higher percentage of detection rate for CV compared to TCBS. The results indicate CV is more specific and accurate than TCBS in detecting *V. parahaemolyticus* (Su and Liu, 2007).

The Wagatsuma agar was developed and used in the Kanagawa Phenomenon. This agar is made up of human or rabbit blood with NaCl, mannitol, crystal violet and K_2_HPO_4_. The main advantage of this agar is to assist in differentiation of *tdh* and non-*tdh* producing strains. *V. parahaemolyticus* strains which produce *tdh* gene will hemolysis a halo on this Wagatsuma agar (Nishibuchi and Kaper, 1995; Qadri et al., 2005; Alipour et al., 2014). The main disadvantage of this media is that the agar cannot differentiate *trh V. parahaemolyticus* strains from the non-pathogenic strains. The *trh* strains will not exhibit hemolysis characteristic on Wagatsuma agar.

### CULTURAL DETECTION AND ENUMERATION

The enumeration of *V. parahaemolyticus* from seafood is important in the context of current FDA guidelines which indicate that shellfish should contain less than 10,000 *V. parahaemolyticus* cells per g (Deepanjali et al., 2005). In line with that, the most probable number (MPN) method was described by U.S. FDA Bacterial Analytical Manual in detecting *Vibrio* species in food samples. MPN is a conventional method that estimates the population density of viable microorganisms in a sample. This method is based upon the application of the theory of probability to the numbers of observed positive growth responses to a standard dilution series of sample inoculum placed into a set of replicate liquid broths (Sutton, 2010). The important perception of the MPN technique is to dilute the sample to an extent that inoculums in the tubes will contain viable organisms. Through replicates and dilution series, the results would be reasonably accurate in estimating the most probable number of cells in the sample. In addition, the nutrient broth used would support growth of organism and turn cloudy. This basic identification step of growth versus no growth provides useful information for low number of organisms (Sutton, 2010). This traditional enumeration method is usually employed during the identification process to identify and enumerate *V. cholerae* and *V. parahaemolyticus* (Nishibuchi, 2006). The FDA has described either a 10-fold, fivefold, or threefold serial dilution MPN tube with selective enrichment broth and agar medium to enumerate *V. cholerae* and *V. parahaemolyticus* in seafood samples (Kaysner and DePaola, 2004). The method involves an overnight enrichment in APW, the standard enrichment for *Vibrio* species detection and then incubated for 18–24 h at 35–37°C. The tubes showing turbidity are cultured on TCBS, a highly selective differential medium that is widely used not only for *V. cholerae* but all other pathogenic *Vibrios* except *V. hollisae* (Kobayashi et al., 1963). In addition, many studies have successfully employed MPN method coupled with selective media CV agar to achieve higher and better isolation of *V. parahaemolyticus* colonies in comparison to the selective TCBS agar (Hara-Kudo et al., 2003; Miyasaka et al., 2006; Blanco-Abad et al., 2009).

Although the conventional detection method is useful in detecting and isolating *Vibrio* species, the method has several major drawbacks. The amount of workload, materials, and the time needed to complete the whole identification process usually takes 7–10 days (Tunung et al., 2011). Hence, to overcome the disadvantages, the MPN method is combined with a species specific polymerase chain reaction (PCR) method. This MPN–PCR method enables the completion of enumeration of bacterial from the environment or seafood samples within 2 days (Miwa et al., 2003; Martin et al., 2004). It is established that the PCR has proven to be very useful because of its ability to amplify a specific DNA segment by a factor of 10^6^ or more within hours, therefore potentially permitting detection of very limited amounts of cells (Alam et al., 2003). Other researchers have reported the success of MPN–PCR method in detecting specific gene in the organism instead of isolation of the target organism to enumerate the bacteria in environmental and food samples (Alam et al., 2002), in soil samples (Vesa et al., 1997) and *V. parahaemolyticus* (Hara-Kudo et al., 2003; Miwa et al., 2003, 2006). The MPN–PCR method can be readily applied using any targeted primer, without extensive developmental work (Luan et al., 2008).

Another alternative approach to the detection and characterization of *V. parahaemolyticus* is via colony hybridization. Colony hybridization technique is a combination of plate count and confirmation based on the identity of the colony through DNA hybridization. Usually most hybridization uses species-specific probes based on variable regions of the 16S rRNA (Thompson et al., 2004). Many studies have described the enumeration using radioactive DNA probes or non-radioactive DNA probes in colony hybridization (Deepanjali et al., 2005). The method was used to confirm the presence and number of total and pathogenic *V. parahaemolyticus* (DePaola et al., 2000, 2003). Suffredini et al. (2014a) proposed that colony hybridization could be a suitable method for the enumeration of total and potentially pathogenic *V. parahaemolyticus* in seafood.

## MOLECULAR DETECTION OF *Vibrio parahaemolyticus*

The conventional phenotyping and biochemical identification techniques of *V. parahaemolyticus* are complicated when the strains are isolated from seafood and aquatic environments (Nishibuchi, 2006). As a result, PCR based assay has become a popular molecular technique for identification and detection of *V. parahaemolyticus* (Drake et al., 2007). The genetic composition of *Vibrio* species is extremely variable thus the genes present inside a targeted strain of *Vibrio* can be used to distinguish the genus from other bacteria and are obvious candidates for the development of DNA based methods for identification of *Vibrio* species (Foley et al., 2009). In fact, a number of researchers have studied pandemic isolates to carry bacteriophage sequences that non pandemic strains do not, and they have exploited these differences to develop pandemic strain–specific detection methods (Bisha et al., 2012). To increase the output and lessen the reagent costs, PCR primers can be multiplexed in a single reaction or tailored for the real-time PCR analysis to provide more rapid results (Grant et al., 2006).

Polymerase chain reaction is a method with high sensitivity and specificity for detection and identification of pathogenic bacteria from clinical, environmental or seafood samples (Nelapati et al., 2012). PCR method was developed to identify *V. parahaemolyticus* strains at the species level by targeting *toxR* gene (Vimala et al., 2010; Paydar et al., 2013; Suffredini et al., 2014b). The *toxR* gene stimulates the expression of *tdh* gene and it is present in either pathogenic or non-pathogenic *V. parahaemolyticus* isolates (Sujeewa et al., 2009). Alternatively, the thermolabile hemolysin (*tlh*) in *V. parahaemolyticus* is another gene that was used to develop a multiplex PCR procedure for simultaneous detection of total and virulent *V. parahaemolyticus* (Yi et al., 2014) Although, the *tlh* gene is not considered a virulence factor of *V. parahaemolyticus*, the gene is reported to be a reliable marker for the bacteria (Su and Liu, 2007). Bej et al. (1999) reported a multiplex PCR protocol for amplification of *tlh*, *tdh*, and *trh*, which could be employed for detecting total and virulent *V. parahaemolyticus* in shellfish. The outcome of results detected *tlh* gene in all 111 strains of *V. parahaemolyticus* isolated from clinical, seafood, environmental, and oyster plants with sensitivity for detecting all three genes of at least 1–10 cells per gram of APW enriched sample homogenate.

Multiplex PCR assays have been very popular and are utilized to differentiate *V. parahaemolyticus*, *V. cholerae*, and *Vibrio alginolyticus* from each other (Di Pinto et al., 2005; Wei et al., 2014). Kaufman et al. (2004) used PCR on samples of oyster mantle fluid, rather than homogenized meat, and reported that *V. parahaemolyticus* levels in the mantle fluid were highly correlated to levels in oyster tissues with *r* = 0.85. Many PCR assays have been employed to affect detection of the *tdh* or *trh* genes (Dileep et al., 2003). In addition, real-time PCR has been used to detect total and pathogenic *V. parahaemolyticus* in seafood samples (Nordstrom et al., 2007). Real-time PCR has the ability to process huge number of samples with speed and consistency in a single tube amplification targeting the gene (McKillip and Drake, 2000). Ward and Bej (2006) developed a multiplexed real-time PCR TaqMan assay that targeted four different genes and was capable of detecting total and pathogenic *V. parahaemolyticus*, including the pandemic O3:K6 serotype in shellfish. The gene targets included the *tdh* and *trh* genes (detection of pathogenic *V. parahaemolyticus*), ORF8 (detection of pandemic *V. parahaemolyticus* O3:K6) and *tlh* gene for the detection of total *V. parahaemolyticus*. Real time PCR was successfully used to detect *tdh* gene (Blackstone et al., 2003) and *tlh* gene (Kaufman et al., 2004) in *V. parahaemolyticus* using specific primer sets and fluorogenic probes.

Current advancement in PCR technology has led to the development of loop mediated isothermal amplification (LAMP) based assays as an alternative to PCR (Notomi et al., 2000). The main advantage of LAMP-based assays as compared to PCR is that during LAMP, nucleic acid amplification occurs at a single temperature, eliminating the need for thermal cyclers. Nemoto et al. (2009) utilized LAMP to detect *tdh*-positive isolates of *V. parahaemolyticus* targeting six regions of the *tdh* gene and compared the results to PCR for detection of *tdh* and reverse passive latex agglutination for *tdh* detection. Another LAMP assay was developed for detection of *tlh* gene and tested both with pure *V. parahaemolyticus* cultures and artificially inoculated shrimp. The assay revealed all 143 pure *V. parahaemolyticus* culture were positive, while no LAMP product was detected from any of 33 non-*V. parahaemolyticus* or 56 non-*Vibrio* isolates (Yamazaki et al., 2008). LAMP consistently identified 2.0 CFU per reaction, while PCR required ∼10-fold more bacteria for detection. Later, Yamazaki et al. (2010) followed up on their previous work by developing a LAMP assay to detect the *tdh* and *trh* genes in *V. parahaemolyticus* and related *Vibrio* species. LAMP assays was also successfully used targeting *rpoD* and *toxR* genes of *V. parahaemolyticus* which resulted in positive detection of 78 *V. parahaemolyticus* strains (Nemoto et al., 2011). The detection LAMP assay sensitivity targeting *rpoD* and *toxR* was determined to be 3.7 and 450 CFU per test in pure culture. The rpoD-LAMP assay was combined with MPN method detection for detection of *V. parahaemolyticus* in spiked short-necked clams comparative to MPN method with a culture method using agar medium. The results showed higher sensitivity using the *rpoD*-LAMP method (Nemoto et al., 2011).

Besides PCR and LAMP assays, there are many other molecular methods (**Table 1**) employed to detect *V. parahaemolyticus* from samples. The random amplified polymorphic DNA–PCR (RAPD-PCR) is another approach commonly used for typing and differentiation of bacteria. This method increases study of genetic relationships between strains and microorganisms, plants or animals species (Oakey et al., 1998). Wong and Lin (2001) used and developed three different PCR methods namely RS-PCR, REP-PCR, and ERIC-PCR to detect *V. parahaemolyticus* to avoid the use of random primers. It was reported REP-PCR is better than ERIC-PCR due to greater reproducibility. Another approach of detecting *Vibrio parahaemloyticus* is through fluorescence *in situ* hybridisation, a method that employs fluorescently labeled short nucleotides to specifically hybridize targeted rRNA in whole permeabilised cells. Sawabe et al. (2009) employed a multi-probe approach (using designed probes VP437, VP612, and VP1253); however, the assay was only species specific, which would only allow for employment of this method to detect total *V. parahaemolyticus*.

**Table 1:** Listof molecular methods for *Vibrio parahaemolyticus* detection.

Methods	Sensitivity/specificity	Advantages/disadvantages	Target and reference
Polymerase chain reaction (PCR)	Sensitivity: Highly sensitive but depends on the PCR program, target separation and enrichment. Specificity: It is highly specific. PCR can be utilized for the detection of total, environmental, pathogenic strains, or even specific serovars.	Advantages: Provides quick, precise, and sensitive results. PCR allows differentiation of pathogenic vs. environmental strains and can be optimized to detect definite serovars. Can be performed as multiplex PCR. Disadvantages: Its sensitivity is hindered by non optimized protocols or enrichments.	*tdh, trh, toxR* (Bhuiyan et al., 2002), *tlh, tdh, trh* (Nordstrom et al., 2007), *tlh, tdh, trh, ORF8* (Ward and Bej, 2006), *toxR, tdh, trh* (Paydar et al., 2013), *tdh, trh* (Suffredini et al., 2014b)
Loop-mediated isothermal amplification (LAMP)	Sensitivity: LAMP is very sensitivity compared to cultural method and even PCR. Specificity: LAMP is less susceptible to interference with 100% specificity. The use of many primers in LAMP provides a greater specificity. Could be used to detect environmental, pathogenic or both strains of *Vibrio parahaemolyticus*.	Advantages: LAMP is simplicity, cost-effective and versatile method which provides rapid results to detect the infected bacteria. Can be performed at one temperature without the need of cycling. Disadvantages: Similar to PCR, it affected by methods of targeted separation and enrichments.	*tlh* (Yamazaki et al., 2008), *tdh* (Nemoto et al., 2009), *tdh*, *trh1,* and *trh2* (Yamazaki et al., 2010), *rpoD* and *toxR* (Nemoto et al., 2011), *Vibrio parahaemolyticus* (Sun et al., 2011), *Vibrio parahaemoluticus tlh* gene (Zeng et al., 2014)
DNA hybridisation	Sensitivity: Compared to cultural method, it is higher sensitive. Specificity: Can be used to detect environmental, pathogenic or both strains of *Vibrio parahaemolyticus*.	Advantages: FDA-BAM suggested method used for *Vibrio parahaemolyticus* identification. Improved rapidity and specificity compared to culture method. Disadvantages: Depends on culture methods which affect the rapidity of detection.	*tdh* (Lee et al., 1992; Nordstrom and DePaola, 2003), *tlh* (Ellison et al., 2001; Gooch et al., 2001)
FISH- fluorescence *in situ* hybridisation and recognition of individual gene fluorescence *in situ* hybridisation (RING_FISH)	Sensitivity: It is a sensitive method applied to detect *Vibrio parahaemolyticus.* Specificity: However, at this point, the differentiation can be done at species level only.	Advantages: Helps to enumerate the number of bacteria in a sample even if low in number. Rapid and specific results. Disadvantages: This method is unable to differentiate pathogenic *Vibrio parahaemolyticus* from environmental isolates. Depends on culture methods which affect the rapidity of detection.	*rRNA* (Sawabe et al., 2009), *tlh* (Griffitt et al., 2011)
Real-Time PCR	Sensitivity: More sensitive than the conventional PCR. It can reduce enrichment step and detect low number of pathogens in a sample. Specificity: The use of fluorescent probes gives the assay a high level of specificity in detecting targeted bacteria from samples.	Advantages: Very efficient, useful, rapid and easy to use in detection of pathogenic Vibrios in seafood. The method does not require post-PCR step. Process by measuring the accumulation of PCR amplicons during each Real Time PCR cycle. The assay could be multiplexed for faster detection. Disadvantages: May amplify dead cells that are not detectable thru cultural methods and amplification could be false positive result.	*gyrB, pR72H, tlh, toxR, tdh,* and *trh* genes (Venkateswaran et al., 1998; Bej et al., 1999; Kim et al., 1999; Davis et al., 2004; Ward and Bej, 2006; Nordstrom et al., 2007; Robert-Pillot et al., 2010; He et al., 2014)

The emergence of a pandemic clone of *V. parahaemolyticus* and its widespread distribution has led to the development of specific method to detect such strains. A group-specific PCR (GS-PCR) based on the sequence variation in the t*oxRS* operon was developed to differentiate between pandemic and non-pandemic strains (Matsumoto et al., 2000). But other researchers claimed that the occurrence of GS-PCR positive *V. parahaemolyticus* strains do not belong to the pandemic clone group (Vongxay et al., 2008). GS-PCR or *orf8-*PCR was developed as a diagnostic tool to identify the pandemic clone group (Nasu et al., 2000).

PFGE of *NotI* digested genomic DNA is a good molecular tool to differentiate between pandemic and non pandemic strains. This method is more appropriate then ribotyping using EcoRI, RAPD-PCR, GS-PCR, and *orf8*-PCR because it produces many diverse patterns and groups the pandemic strains in closely related clusters (Yeung et al., 2002). Many studies have utilized this method and validated the reproducibility and discriminatory nature of PFGE (Fakruddin et al., 2013). PFGE is also able to produce results of the genetic diversity among strains which is important information that is not provided by GS-PCR or *orf8*-PCR. A few studies have stated that repetitive sequenced based PCR is found to be slightly more discriminatory compared to PFGE as it generates greater numbers of different patterns and was less likely to yield untypeable results caused by DNA degradation (Wong and Lin, 2001).

## CONCLUSION

The concerns about health consequences from *Vibrio* species, especially when seafood remains as a vehicle of transmission of *Vibrio*, are likely to continue in future. Over the last decade, at least one new *Vibrio* species has been reoccurring per year that could be transmitted through the environment as a new public health threat. This is due to a number of factors including: (i) progress in molecular biology, which allows identification of new strains and locates its source; (ii) the evolution of pathogens; and (iii) application of microbial risk assessment to quantify risks from environmentally transmitted pathogens (Igbinosa and Okoh, 2008). Therefore, to establish effective control measures to reduce the risk infection by this bacterium and to ensure the safety of foods; surveillance and epidemiology as well as the employment of molecular methods for the detection of *V. parahaemolyticus* in food and environment is very important. Phenotypic and molecular detection will continue to be useful in isolating and identifying of *V. parahaemolyticus* as this bacteria continues to emerge as a food borne pathogen.

## Conflict of Interest Statement

The authors declare that the research was conducted in the absence of any commercial or financial relationships that could be construed as a potential conflict of interest.
